# Dr. Sigmond on the Treatment of Idiots

**Published:** 1848-01-01

**Authors:** G. G. Sigmond


### THE IDIOTS OF THE BICÊTRE.

BY G. G. SIGMOND, M.D.

MANY essays have been lately published on the subject of the extraordinary strides which are being made towards the conversion of the idiot into the man fitted for the ordinary occupations of society. All the publications, however, which have as yet appeared, have proceeded from authors who are recognised in the fields of imagination as legitimate exaggerators of all they see and hear, and are permitted to draw largely on their fancy for illustrations of the pictures they present. At length, we are furnished with something in a more authoritative shape, and the observations of a man of science and of acknowledged character are placed before us in a proper form. Upon M. Brierre de Boismont it devolved to give a resumé of the labours of Séguin for the "Annales d'Hygiene Publique;" and previously to his undertaking the task, he determined personally to investigate the present state of the idiots in the Bicêtre, and the result of his experience he has given to the medical profession. His visit was evidently undertaken to ascertain how far the report drawn up by Messrs. Serres, Flourens, and Pariset, in favour of Séguin's method, was borne out by the facts; and, without throwing any censure upon the directors of the Bicêtre, he seems to have learnt with surprise that the means pursued at the Bicêtre had no connexion whatever with those of M. Seguin, which have attracted so much attention. We will not discuss the claims of France to evincing stronger feelings of humanity than any other nation, nor follow M. Brierre de Boismont through the splendid eulogiums which he has passed on his native land; but we must remind him that Itard and Pinel, to whose labours he pays a proper compliment, forget not to point to Willis and Crichton of England as the founders of the system of moral and of humane treatment for those unfortunate beings whom modern science has taught us to treat with gentleness and with sympathy. As we hope to have the opportunity of elsewhere considering both Séguin's "Hygiene et Education des Idiots," and likewise his recent energetic protest in favour of Perere, as deserving the honours we now give to the Abbé de l'Epée, we shall confine ourselves to M. Brierre de Boismont's description of the scenes which offered themselves to him when he was admitted to the school of idiots. This portion of the institution is under the management of Dr. Voisin, and has for the last three years been under the more immediate superintendence of M. Vallée, who, accompanied by M. Mallon, and three American physicians, paid every attention to the wishes of the investigator, and explained to him the views which were entertained by the administration of the establishment. M. Mallon, the director, observed, "Our object is to bring into action the best part of that which remains to these helpless beings of motive power, and of their moral and intellectual faculties." Acquainted with the mechanism which too often attends exhibitions intended to cajole individuals in favour of institutions, M. Brierre de Boismont was soon interested in all he saw, and laid aside all those suspicions which at first naturally take possession of the mind, and lead to doubts of the sincerity of those who guide, and of the reality of the benefits which are said to be produced.

The class consisted of about fifty idiots, amongst whom were about a dozen epileptics, who, by their superior intelligence, served as monitors. M. de Boismont passed them in review with great care; the stamp of idiocy was on the face of each, varying certainly in its intensity, but indisputable. They were ranged in a semicircle in face of the investigators. They stood all calmly upright, making no disorderly movement, and each retaining his rank. They were of all ages, from six up to eighteen. Their appearance bespoke health. During a whole hour, whilst they were under examination, they committed not the slightest act of indiscretion, showed no want of discipline, nor made use of any improper expression. The first exercises consisted of singing, in which all but three or four took part. The intonations were correct, and all in harmony. The pieces consisted of hymns, and the orchestra was composed of blind persons. A little child of six years of age, only a few months in the establishment, who had been brought in in a most deplorable condition, scarcely able to pronounce, and kept with difficulty in any one posture, sang with feeling and propriety three stanzas, which were repeated by the other idiots in chorus. This child afterwards repeated with distinctness, and with well marked emphasis, the fable of the Wolf and the Lamb. Dancing was executed with great precision and even elegance by three of the children, whilst the other idiots looked on with attention. One of the assistant-masters made three other idiots go through the military exercise without the commission of the slightest mistake. The copy-books of the children were exhibited in a frame; they were clean, well kept, and so correct as to bear comparison with those of the most intelligent children. There was one idiot whose appearance particularly struck our author; he was paralytic, squinted, and his mouth wide open, his tongue rolling out of his mouth; he articulated very indistinctly, he dragged himself upon his knees, and his hands were deformed. His writing, nevertheless, was very legible in large characters, and it was kept very clean. That no doubt could be entertained of the identity, M. Vallée placed him near the table, and put before him a slate, on which, after writing the name of M. de Boismont, he desired the idiot to copy it. This was done in a legible hand. It is necessary, says the examiner, to have seen the face, the gestures, and this type of idiocy to form an idea of what patience, what perseverance must have been bestowed to obtain such a result. This same idiot played a game of dominoes with Dr. Fisher, one of the American gentlemen. His exultation on taking a domino was very striking. The first game going against him, he exhibited evident marks of discontent; and when his adversary placed his last domino at the end of the board, the poor idiot threw himself at the back of the chair, exhibiting his desire to be taken away; but when the American physician offered him his revenge, he expressed the greatest satisfaction, which was extreme indeed, when he found that he had gained the victory. The instructor then placed before him a copy of several words, and a case where the letters of the alphabet were arranged, and then asked him to imitate one of the words which he pointed out. The poor idiot, rubbing his hands with pleasure, looked out the letters successively, and composed the word required. M. Vallée then called forward an idiot of about fifteen years of age, and requested M. de Boismont to dictate a phrase. He wrote it out immediately without making the slightest error in orthography, and with all the capital letters correct but one. On his being simply asked if the whole was correct, he effaced the small letter to place a large one. After some short time, the gymnastic exercises followed upon the intellectual ones. Two children swung themselves by their feet. Five idiots placed in a line, and who had just commenced their education, drew out, one after the other, the different parts of the face, as they were pronounced by the master in a loud voice; they made but few mistakes. One idiot particularly arrested the attention of M. de Boismont; he was between fifteen and sixteen; the absence of expression in his eyes, the movements of his head, his stuttering speech, his whole appearance bespeaking him to be one of the most characteristic types of the class of idiots. The director called for the book describing him at the period of his admission, which was found to be the month of June, 1843.

He was there described as being incapable of articulation, or of remaining tranquil for one instant, executing the most irregular movements and the most disgusting actions, urged on by instinct to self-destruction, having but one redeeming quality, the desire of approbation; indeed, the picture drawn of him at the time of his admission can scarcely be given. At the invitation of M. Vallon, he named all the geometric figures, formed of pasteboard, which were placed in his hands, he drew them upon paper with the assistance of a rule and compass; this was accompanied by some hesitation; then he repeated several times the name. Upon a bandage being applied to his eyes, he named the different forms presented to him. A small chest of drugs, containing twenty different substances in rows, was placed before him; he named each article that was presented to him when uncorked; he paused a little when the odours were not very powerful, but ether and ammonia were in a moment recognised; the taste served him as a guide to some; when the bandage was again applied, he distinguished the substances, with very few mistakes. A card on which different colours were painted was shown him, and he pointed them out correctly; a series of figures he counted when there were not more than two, but he hesitated when there were three, the units and the tens he fully comprehended, but when the amount was hundreds he paused. Another idiot of fourteen added and multiplied with great exactness. The mental operations of some of the individuals were accomplished by considerable labour; thus, one of them having been asked to divide twelve counters which were given to him into half, first ranged them all in one long column, then taking them one after the other away from the top placed them side by side until two equal columns were formed, then stopped, and pointed out that his labour was completed; upon being asked to divide them into four, he again placed them in one long column, and taking three from the top placed them side by side of the original column, till he had made his four equal lines, and then again pointed with his finger to what he had accomplished. M. Vallée pointed out one of the idiots who had the most extraordinary facility for calculation, and who performed the most complicated multiplications instantaneously. His mental operation was to multiply the units, the tens, and the hundreds separately, and then to add the whole result. Upon being asked to multiply sixty-four by forty-two he gave the product, 3968, before the master had time to calculate with his pencil. After this exhibition, the party of investigators went into a hall where were exhibited about thirty drawings, well executed; and there were placed, each following some avocation, as a joiner or a shoemaker, most of the idiots they had so lately examined. Among the joiners was the idiot who had excited so much attention; he was polishing with great care a large board; some were making small pieces of furniture, others window-blinds; everything was made by themselves; they were permitted to use scissors, planes, vices, presses, and no accident had occurred. The greatest silence was preserved in this shop, as well as in that of the shoemakers. They took up several pieces of leather, which they had sewed with great exactness, the ends being in perfect straight lines; more than sixty pairs of shoes made by them were shown. Some of them were fond of agricultural pursuits, and in fine weather employed themselves in work out of doors. It was remarkable, that although attentive to all that passed, they scarcely spoke, and the same isolated state was observable in the workshops. The ideas, however, are so limited amongst idiots, that the necessity of communicating them is scarcely felt.

M. de Boismont concludes with a well-merited compliment to Messrs. Vallée and Mallon for the part they have taken in this interesting experiment, and for the care they have bestowed upon these unfortunate beings. By dint of patience, of determination, of capacity, poor idiots have arrived at the power of reading, writing, calculating, drawing, but that which is more important still, they have learned to work. It is, however, to be borne in mind, that the master, the teacher, is always at their side, directing them by gesture, by voice, by look—that, in fact, he is their leading-file. But still there can be no comparison between them and the idiot chuckling, bawling, howling, incapable of every sort of labour, deprived of every moral sentiment, having only animal instincts, and there can be no doubt that some of these individuals will gradually be raised above that miserable condition to which they were apparently destined; and as science extends her domains, and unites with the spirit of humanity and benevolence, there will be rescued from their deplorable state, souls to which may be extended the hopes, the comforts, and the expectations of eternal happiness, which spring from the truths of religion. Useful certainly are these strictures of M. de Boismont, and most sincerely do we desire that he may be led by what he has seen still farther to inquire into the development of the senses, and to examine strictly into the works of the experienced men who are cultivating this new field of inquiry, so that we may have in those archives which are becoming so important to medical science, a faithful review of the labours which are now prosecuting in France, and from which we have a right to expect so much: if, as he imagines, his predecessors have left behind them richer treasures than are to be found in other countries, so much more does it behove them to whom power is now delegated to scrutinize that which is passing before them with earnest zeal, and with a determination to support that which promises to be most useful to man, if he is perfectly satisfied that the system which is proposed by Séguin is superior to all others, he is acting an honest part to uphold it in preference to all others; but he must remember that, as his opinion will carry weight with the scientific world, he must not hastily pronounce a judgment that he is not prepared to uphold during all the stages of inquiry.

